# The Marriage of the Unfit

**Published:** 1898-09-17

**Authors:** 


### THE MARRIAGE OF THE UNFIT.

Dr. Harry Campbell,[1] writing on the marriage of the unfit, would very considerably extend the boundaries of that group of diseases which go by the name "hereditary," and would thus increase in no small degree the number of those who, for the benefit of the race, ought to be held unfit for marriage. He shows how largely natural selection is interfered with among civilised communities—on the one hand, by a continual saving of lives which in more natural conditions would succumb, and, on the other, by the abolition of polygamy. Putting polygamy, however, more or less regretfully on one side as a means of rejuvenating the race, and taking the institution of marriage as it stands, the question is, Who should marry? or, perhaps, more properly, Who should refrain from marriage?

Into the latter category Dr. Harry Campbell places at once all sufferers from pulmonary consumption, organic heart disease, epilepsy, insanity, diabetes, and chronic Bright's disease, and also from rheumatic fever, if there has been more than one attack, as obviously unfit. To these he adds all those cases of non-accidental disease in which life is saved by the surgeon's skill, cases which in a less artificial condition of existence would be weeded out by natural selection and thus prevented from transmitting to others a like defect. "Think," he says, "of the millions and millions of women suffering from ovarian cyst whom nature has in the past sacrificed with a cruel kindness in order to keep a certain level of ovarian fitness in the race. These are practically all saved now in civilised communities, with the result that the percentage of ovarian cysts is necessarily increasing. It can only be that medical men do not realize the potency of heredity that they sanction the propagation of children by women whose lives have been saved in this manner."

[1] Lancet, Sept. 10.

The class of disorders, however, which Dr. Campbell thinks "should beyond almost all others be regarded as a bar to marriage" are functional disorders of the nervous system. The highly sensitive, he says, are not suited for this hard world; its strenuous conditions call for men of iron nerve and stout hearts, and, as regards happiness, the fine animal with little imagination and a good thick skin has the best time of it—in other words is the most "fit."

It is pointed out that the lives of people with weak nerves are often one long drawn out misery, and to the neurotic man the advice is given "Remain single." As a counsel of perfection this may be right enough, and it may perhaps, be true that a world of stolid thick-skinned mediocrities offers the greatest happiness for the greatest number. Nevertheless we look with some regret at the idea of a world without neurotics. In one of the most brilliant lectures ever delivered before the Royal College of Physicians, Professor Clifford Allbutt said, "Why, gentlemen, my neurotic patients are almost the best people in this wicked world! Rarely endowed with the capacity, endurance, and profounder imagination of the greatest, they form a large number of those in the second rank who are the salt of the earth." Yet, perhaps, if the happiness of posterity is to be looked upon as our end and aim, even the neurotics had better refrain from marriage, for, great as are their mental aptitudes, their capacity for sinking into the depths of wretchedness is undoubted—and this also is hereditary.

